# Comparison of Pregnancy Outcome between 4 and 6 cm Cervical os Dilatation to Demarcate Active Phase of Labour: A Cross-Sectional Study

**DOI:** 10.1155/2023/8243058

**Published:** 2023-06-26

**Authors:** Nadzirah Mohd Fathil, Rahana Abd Rahman, Azmawati Mohd Nawi, Ixora Kamisan Atan, Aida Hani Kalok, Nor Azlin Mohamed Ismail, Zaleha Abdullah Mahdy, Farin Masra, Zuhailah Muhammad, Shuhaila Ahmad

**Affiliations:** ^1^Department of Obstetrics & Gynaecology, Faculty of Medicine, Universiti Kebangsaan Malaysia, Kuala Lumpur, Malaysia; ^2^Hospital Canselor Tuanku Muhriz, Jalan Yaacob Latif, Bandar Tun Razak, Kuala Lumpur, Malaysia; ^3^Department of Community Health, Faculty of Medicine, Universiti Kebangsaan Malaysia, Kuala Lumpur, Malaysia; ^4^Department of Paediatrics, Faculty of Medicine, Universiti Kebangsaan Malaysia, Kuala Lumpur, Malaysia

## Abstract

This is a cross-sectional study comparing pregnancy outcomes between participants with 4 and 6 cm of cervical os dilatation at the diagnosis of the active phase of labour. It was conducted in a single tertiary centre involving low-risk singleton pregnancies at or beyond 37 weeks with spontaneous onset of labour. A total of 155 participants were recruited, 101 in group 1 (4 cm) and 54 in group 2 (6 cm). Both groups were similar in mean maternal age, mean gestational age at delivery, ethnicity, median haemoglobin level at delivery, body mass index, and parity. There were significantly more participants in group 1 who needed oxytocin augmentation (*p* < 0.001) for the longer mean duration (*p* = 0.015), use of analgesia (*p* < 0.001), and caesarean section rate (*p* = 0.002). None of the women had a postpartum haemorrhage or a third- or fourth-degree perineal tear, and none of the neonates required admission to the neonatal intensive care unit. There were significantly more nulliparas who had a caesarean section as compared to multiparas. A cervical os dilatation of 6 cm reduces the risk of caesarean section by 11% (95% CI, 0.01-0.9) and increases three times more the need for analgesia (AOR = 3.44, 95% CI, 1.2-9.4). In conclusion, the demarcation of the active phase of labour at a cervical os dilatation of 6 cm is feasible without an increase in maternal or neonatal complications.

## 1. Introduction

Traditionally, labour can be divided into 3 stages, and the first stage is further subclassified into latent and active phases. A cervical dilation of 4 cm marks the beginning of the active stage of labour. This is the rapid, accelerated phase of labour, in which nulliparas and multiparas are projected to have cervical dilatation rates of 1 cm/hour and 0.5 cm/hour, respectively [1].

The World Health Organization (WHO) recommended a caesarean section rate of 10-15% [2]. However, the rise beyond the recommended rate had been a major concern and led to an increase in research and debate amongst healthcare professionals, governments, and clinicians. Globally, the caesarean rate has increased to as high as 40.5% in Latin America and the Caribbean region [3]. Likewise, Asian countries are facing similar issues, with both medical and social factors contributing to the surge [4].

In Malaysia, there is a similar rising trend ranging from 18.8 to 31.5% in 12 different government-funded hospitals [5]. Based on a study looking at the caesarean section rate in 12 Malaysian tertiary hospitals using Robson's 10-group classification, the major contribution to the overall caesarean section rates every year was from Robson's groups 1 (nulliparous, singleton, cephalic, ≥37 weeks, spontaneous labour) and 3 (multiparous women, singleton, cephalic, ≥37 weeks, without a previous caesarean scar, spontaneous labour). Numerous measures had been undertaken to reduce caesarean section rates. However, there has not been a significant reduction, which is most likely owing to a combination of factors. The decision to perform a caesarean section is influenced by the different practices of various hospitals as well as individual obstetricians.

The caesarean section carries inherent risks of mortality and morbidity for both the mother and the neonates. Previous caesarean delivery is associated with a higher risk of uterine rupture, morbidly adherent placenta, placenta praevia, and severe maternal outcome. Likewise, there is an increased risk of early neonatal death, preterm birth, and neonatal intensive care unit (NICU) admission [6]. The higher risk of NICU admission is probably due to the higher risk of respiratory complications in neonates delivered by elective caesarean section [7].

There is an urgent need to scrutinize and change our intrapartum management of low-risk women especially those without caesarean scars. The objective of this study is to compare the maternal and perinatal outcomes of women who were diagnosed to be in the active phase of labour at 4 cm versus 6 cm cervical os dilatation.

## 2. Materials and Methods

This was a cross-sectional prospective study from 1st May 2021 to 31st December 2021 in a single tertiary centre. It is located in Kuala Lumpur, the capital city of Malaysia, with deliveries of 4000 to 6000 per year. Institutional review board approval was obtained on 1st May 2021 from the hospital ethics committee (FF-2021-121). Written informed consents were obtained from each patient. All methods performed were following the relevant guidelines and regulations. The research had been performed in accordance with the principles stated in the Declaration of Helsinki.

All low-risk participants who were aged 18 years old and above with no medical disorders such as diabetes and hypertension, fetal complications such as small for gestational age, fetal growth restriction, oligohydramnios, polyhydramnios, singleton normal fetus, admitted in spontaneous labour at 37 weeks gestation or more, and cervical dilation of 4 and 6 cm at diagnosis of the active phase of labour with the cephalic presentation were recruited. The clinical assessment was performed by the attending doctor either in the ward or in the admission centre. The attending doctors have three to five years of experience working in the O&G department and are undergoing training in the masters program. Exclusion criteria were women who had labour induction, previous uterine scars, and a prepregnancy body mass index (BMI) of more than 30 kg/m^2^. Upon obtaining the written informed consents, an amniotomy will be performed, and labour is managed as per standard protocol.

Data on maternal demography and clinical characteristics such as maternal age, gestational age at birth, ethnicity, haemoglobin level at birth, prepregnancy BMI, and parity were collected. Prepregnancy weight was obtained from the patient's self-report. BMI is classified based on WHO, i.e., <18.5 kg/m^2^ as underweight, 18.5-24.9 kg/m^2^ as normal, and 25-29.9 kg/m^2^ as overweight [8]. Intrapartum management details and pregnancy outcomes such as meconium stained liquor, oxytocin augmentation, mean duration of the second stage, use of analgesia, mean duration from diagnosis of the active phase of labour to birth, caesarean section rate, postpartum haemorrhage, third- and fourth-degree perineal tear, birth weight, Apgar score at 5 minutes, arterial cord pH at birth, and requirement for neonatal intensive care unit (NICU) admission were recorded.

The sample size of those at 4 and 6 cm cervical dilatation was 3 : 1. The sample size was calculated using http://openepi.com/SampleSize/SSCC.htm. At a 95% confidence level and 80% power, the sample needed was 155 women. Based on 1 : 3 ratio, a total of 54 women are needed for the 4 cm cervical dilatation group, and 104 women are needed for the other group. The 1 : 3 ratio is due to the difficulty to obtain women with 6 cm cervical os dilatation at the diagnosis of the active phase of labour. Data entry and statistical analysis were performed using the Statistical Package for Social Sciences (SPSS) software (version 25.0). The relationship between study variables was analyzed using appropriate statistical analysis. The mean and standard deviation were used for continuous variables, while frequency and percentages were used for categorical variables. The independent *t*-test was used to ascertain the significance of differences between the mean values of two continuous variables with a normal distribution. The chi-squared or Fisher's exact test was used to determine the associations between individual categorical independent factors and outcomes. The value of *p* < 0.05 was considered significant. The relationships between variables and comparability of groups (4 cm versus 6 cm) with the outcome of interest (maternal and fetus) were tested individually using linear regression.

## 3. Results

**Figure 1:**
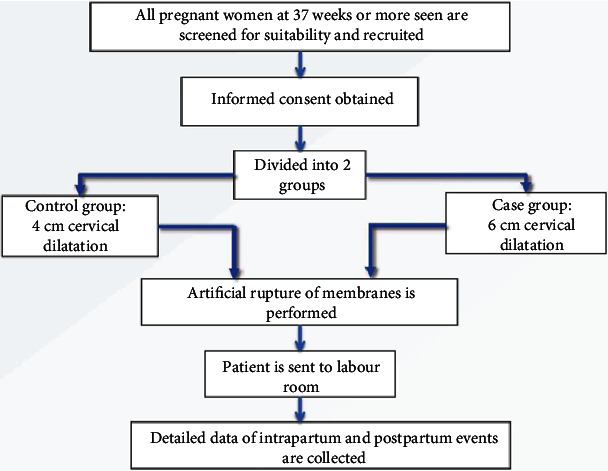
Patient recruitment and flow chart of the study.

A total of 155 parturient who met the inclusion criteria were recruited, with 101 participants in group 1 (4 cm cervical os dilatation) and 54 in group 2 (6 cm cervical os dilatation) (Figure 1).

Table 1 demonstrates the demographic data compared between the two groups. The majority of our participants are Malay, with age of less than 35 years old. Both groups are similar in mean maternal age, mean gestational age at birth, ethnicity, median haemoglobin level at birth, median prepregnancy BMI, and parity.

Table 2 compares the intrapartum management and pregnancy outcome between the two groups. There is a significantly lower caesarean section rate (*p* = 0.002), lesser use of oxytocin infusion for augmentation (*p* = <0.001), shorter mean duration of oxytocin infusion (*p* = 0.015), lesser use of analgesia (*p* < 0.001), and shorter duration from diagnosis of the active phase of labour to birth (*p* < 0.001) in group 2 as compared to group 1. The two groups are similar in the rate of meconium-stained liquor, mean duration of the second stage, caesarean section rate for fetal distress and poor progress of labour, mean birth weight, and arterial cord pH at birth. None of the participants had postpartum haemorrhage or sustained third- or fourth-degree perineal tear, and all the neonates delivered had an Apgar score of more than 7 at 5 minutes and did not require NICU admission (not shown in the table).

Table 3 shows a comparison of demographic data between participants who delivered via caesarean section and vaginal delivery. Both groups are similar in mean maternal age and median prepregnancy BMI. There are significantly more nulliparous participants who had caesarean deliveries as compared to multiparous participants (*p* = 0.023).

Table 4 shows a multivariable analysis to determine the relationship between the caesarean section and other confounding factors. A cervical os dilatation of 6 cm reduces the risk of caesarean section by 11% (95% CI, 0.01-0.9) and increases three times more the need for analgesia (AOR = 3.44, 95% CI, 1.2-9.4).

## 4. Discussion

To date, the exact demarcation of the active phase of labour is still debatable. Every woman has different and unique durations of labour. Due to this wide variation, it has been difficult to accurately determine the normal duration of the latent and active phases of labour. Likewise, the exact timing of the transition from the latent to active phase is not clear. The worry of changing the demarcation of the active phase of labour is the maternal and neonatal outcomes. The ultimate aim of the change is to reduce the caesarean section rate but without jeopardising both the mother and neonate. Hence, for the past few years, many researchers had conducted clinical trials to answer this question.

The original partogram created by Friedman had demarcated that accelerated cervical dilatation occurs around 3 to 6 cm [1]. It is interesting to discover that as early as 1986, an attempt to investigate the actual transition from latent to active phases of labour was done in the US, involving 1699 women retrospectively [9]. It was acknowledged that at 4 cm cervical dilatation, the progress is slow, and this is probably because this is still the latent phase of labour. Only at 5 cm, nearly 90% of women demonstrated an increasing rate of cervical dilatation. Therefore, the concept of labour progress at 1 cm per hour is not applicable. Another retrospective study done in Japan in 2015 involving 2369 women demonstrated different trends of labour from Friedman. The progress from 4 cm to 10 cm takes about 5 hours, and the rate of dilatation follows 1 cm per hour only at the late stage, starting at 8 cm [10]. About 56 years later, this has been challenged by Zhang et al. who found that labour progresses slower before 6 cm cervical dilatation and had suggested that the demarcation of the active phase of labour should be changed as the results of Friedman could not be replicated [11]. Our participants were a mixture of both nullipara and multipara. In agreement with the previous studies, we demonstrated that at 6 cm cervical os dilatation, both parities progressed significantly faster [11–13].

The main finding of our study was the significantly lower caesarean section rate in participants who were diagnosed in the active phase of labour at 6 cm cervical os dilatation. This reduces the risk of caesarean delivery by 11% as compared to 4 cm. In addition, there were no apparent adverse events to bother the mother and neonate. This was a prospective observational study which concurred with other similar study designs that had been published. A study done in France by Thuillier et al. involved a larger cohort of women comparing between before (*n* = 3283) and after (*n* = 3068) modification of the demarcation of the active phase of labour. Similarly, they included only low-risk women and demonstrated a reduction of the caesarean section rate from 9.4% to 6.9% with similar maternal and neonatal outcomes between the two groups [14]. In contrast, a study done by Rosenbloom et al. had shown different results despite similar characteristics of patients. There was no reduction in the primary caesarean section rate despite an increase in maternal and neonatal morbidities [15].

The reduction in the caesarean section rate was found in other published data involving low-risk women with no uterine scars [12, 14, 16, 17]. Wilson-Leedy et al. performed a retrospective cohort study before and after the change of demarcation of the active phase of labour. All nulliparous participants without uterine scars who underwent induction or augmentation of labour were included. The change of the active phase of labour to 6 cm cervical os dilatation has led to a significant reduction in the caesarean section rate from 35.5% to 24.5% [18]. In line with the aim to improve labour management, WHO had also changed the demarcation of the active phase of labour to 5 cm cervical dilatation in 2018 [19].

To our knowledge, this is the first prospective study in Malaysia to investigate the feasibility and pregnancy outcomes if the demarcation of the active phase of labour is changed from 4 to 6 cm. We recently had published our retrospective study comparing the outcomes of women with cervical dilatation of 4 versus 6 cm. We were able to demonstrate a significant reduction in caesarean section rate with fewer neonatal complications [20]. Although this current study is underpowered with a nonequal ratio between the two groups, we did not find any association with increased rates of adverse maternal or neonatal outcomes. A cervical os opening of 6 cm has a significant protective effect from caesarean section, but the confidence interval is wide which is most likely due to the small sample size. It is also important to highlight that in our centre, we managed labour actively. Routine amniotomy is performed for all women diagnosed with the active phase of labour with intact membranes. Therefore, we are not able to know whether the results of this study were influenced by this practice. To date, WHO and a few other international guidelines do not recommend routine amniotomy [19, 21]. However, this is practised in our centre due to the logistic issues.

A larger clinical trial is needed to confirm the findings of our study. We have recently changed the labour management policy by changing the demarcation of the active phase of labour from 4 to 6 cm cervical dilatation for low-risk women. There will be an opportunity to look at the impact of this modification which will involve a larger sample size.

## 5. Conclusion

Redefining the active phase of labour from 4 to 6 cm is feasible and is associated with a reduced caesarean section rate without significant adverse maternal and neonatal outcomes amongst low-risk women.

## Figures and Tables

**Table 1:** Demographic data of study population comparing between 4 cm and 6 cm cervical os dilatation, *n* = 155.

	Cervical dilatation		*p* value
	Group 1 (4 cm) *n* = 101	Group 2 (6 cm) *n* = 54	
Mean maternal age ± SD, years	30.4 ± 4.5	31.2 ± 4.4	0.284^∗^
Group age (years), *n* (%)			0.833^∗∗^
<35	82 (81.2)	43 (79.6)	
≥35	19 (18.8)	11 (20.4)	
Mean gestational age ± SD, weeks	39.1 ± 1.0	38.9 ± 0.9	0.285^∗^
Ethnicity, *n* (%)			0.421^∗∗^
Malay	85 (84.2)	48 (88.9)	
Non-Malay			
Chinese	9 (8.9)	4 (7.4)	
Indian	1 (1.0)	—	
Others	6 (5.9)	2 (3.7)	
Median haemoglobin (IQR) level at delivery (g/dL)	12.1 (11.4-12.6)	12.3 (11.5-13.0)	0.197^∗∗∗^
Median prepregnancy BMI (IQR) (kg/m^2^)	26.0 (24.1-27.9)	25.6 (23.2-28.3)	0.613^∗∗∗^
BMI category, *n* (%)			0.123^∗∗∗∗^
Underweight	0 (0.0)	2 (3.7)	
Normal	33 (32.7)	20 (37.0)	
Overweight	68 (67.3)	32 (59.3)	
Parity, *n* (%)			0.109^∗∗^
Nulliparous	35 (34.7)	12 (22.2)	
Multiparous	66 (65.3)	42 (77.8)	

^∗^Independent *t*-test, ^∗∗^Pearson's chi-square test, ^∗∗∗^Wilcoxon's rank sum test, ^∗∗∗∗^Fisher's exact test. SD = standard deviation; BMI = body mass index; IQR = interquartile range.

**Table 2:** Intrapartum management and labour outcomes between women with cervical os dilatation of 4 cm and 6 cm, *n* = 155.

	Cervical os dilatation		*p* value
	Group 1 (4 cm) *n* = 101	Group 2 (6 cm) *n* = 54	
Meconium-stained liquor at amniotomy, *n* (%)	14 (13.9)	5 (9.3)	0.405^∗^
Oxytocin augmentation, *n* (%)	51 (50.5)	8 (14.8)	<0.001^∗^
Mean duration of augmentation ± SD, min	164.1 (98.4)	75.0 (48.1)	0.015^∗∗^
Median duration of second stage (IQR), minutes	8.4 (4-10.5)	6.8 (3-8)	0.167^∗∗∗^
Use of analgesia, *n* (%)	27 (26.7)	1 (1.9)	<0.001^∗∗∗∗^
Epidural	24 (88.9)	1 (100)	
Pethidine	3 (11.1)	0	
Median duration from amniotomy to delivery (IQR), minutes	217 (120-300)	95.7 (30-142.5)	<0.001^∗∗∗^
Caesarean section, *n* (%)	20 (19.8)	1 (1.9)	0.002^∗^
Caesarean section for fetal distress, *n* (%)	15 (14.9)	1 (1.9)	0.762^∗∗∗^
Caesarean section for poor progress, *n* (%)	4 (4.0)	0 (0.0)	0.810^∗∗∗^
Mean birth weight ± SD, gram	3112 ± 384	3093 ± 398	0.768^∗∗^
Arterial cord pH ≤7.20, *n* (%)	8 (9.1)	2 (4.2)	0.494^∗∗∗∗^

^∗^Pearson's chi-square test, ^∗∗^Independent *t*-test, ^∗∗∗^Wilcoxon's rank sum test, ^∗∗∗∗^Fisher's exact test. SD = standard deviation; IQR = interquartile range.

**Table 3:** Comparison of demographic data between participants who delivered via caesarean section (*n* = 21) and vaginal delivery (*n* = 134).

	Vaginal delivery *n* = 134	Caesarean section *n* = 21	*p* value
Mean maternal age ± SD, years	30.9 ± 4.6	29.3 ± 3.9	0.125^∗^
Group age (years), *n* (%)			0.767^∗∗^
<35	107 (79.9)	18 (85.7)	
≥35	27 (20.1)	3 (14.3)	
Median prepregnancy BMI (IQR) (kg/m^2^)	26.4 (24.0–28.2)	26.3 (24.0–27.6)	0.924^∗∗∗∗^
BMI category, *n* (%)			0.311^∗∗^
Underweight	1 (0.7)	1 (4.7)	
Normal	47 (35.1)	6 (28.6)	
Overweight	86 (64.2)	14 (66.7)	
Parity, *n* (%)			0.023^∗∗∗^
Nulliparous	36 (26.9)	11 (52.4)	
Multiparous	98 (73.1)	10 (47.6)	

^∗^Independent *t*-test, ^∗∗^Fisher's exact test, ^∗∗∗^chi-square test, ^∗∗∗∗^Wilcoxon's rank sum test. SD = standard deviation; BMI = body mass index; IQR = interquartile range.

**Table 4:** Multivariate analysis on the factors associated with caesarean section (*n* = 21).

Variable	Simple logistic regression		Multivariate logistic regression	
	OR (95% CI)	*p* value	AOR (95% CI)	*p* value
Oxytocin augmentation (yes)	2.91	0.027	0.94 (0.3-3.6)	0.926
Use of analgesia (yes)	5.5	0.001	3.44 (1.2–9.4)	0.017
Cervical dilatation at amniotomy (6 cm)	0.01	0.076	0.11 (0.01–0.9)	0.040
Duration from amniotomy to delivery, hours	1.00	0.001	1.00 (0.9–1.0)	0.361

OR = odds ratio; AOR = adjusted odds ratio; CI = confidence interval.

## Data Availability

The data used to support the findings of this study are restricted by the Ethics Committee of UKM Medical Centre in order to protect patient privacy. Data are available from Rahana Abd Rahman, drrahana@ppukm.ukm.edu.my, for researchers who meet the criteria for access to confidential data.
